# Intraoperative hemodynamics and anesthetic implications in superobese parturients undergoing cesarean delivery: a retrospective cohort analysis

**DOI:** 10.1007/s00404-026-08408-0

**Published:** 2026-04-04

**Authors:** Teshi Kaushik, Andrew Hackney, Ayesha Bryant, Elizabeth Baker, Sijules Abongwa, Brant M. Wagener, Michael Arnold Frölich

**Affiliations:** Department of Anesthesiology and Perioperative Medicine, University of Alabama at Birmingham, Heersink School of Medicine, Birmingham, AL USA

**Keywords:** Maternal obesity, Cesarean section, Neuraxial anesthesia, Hypotension

## Abstract

**Background:**

Obesity affects nearly 40% of reproductive-aged American women, with over 12% classified as severely obese. Higher maternal body mass index (BMI) is linked to increased cesarean rates; however, data on intraoperative hemodynamics and anesthetic management, particularly in the superobese (BMI ≥ 50 kg/m^2^), remain limited.

**Methods:**

We conducted a retrospective cohort study of 2051 cesarean deliveries under neuraxial anesthesia, stratified by six BMI categories. The primary outcome was intraoperative hypotension based on serial noninvasive blood pressure (NIBP) readings. Secondary outcomes included neuraxial placement time, surgical duration, estimated blood loss (EBL), and neonatal Apgar scores. Multivariate regression, adjusted for comorbidities and neuraxial technique, assessed associations and outcome differences across BMI groups.

**Results:**

Superobese parturients exhibited a delayed yet sustained decline in mean arterial pressure (MAP) following neuraxial anesthesia, showing a late-phase, vasopressor-resistant hypotensive pattern. Time from surgical procedure start to fetal delivery increased from 11.8 ± 9.1 min in normal weight versus 17.3 ± 9.3 min in superobese (*p* < 0.0001). Between BMI groups, anesthesia start to procedure start, which reflected positioning, IV optimization, and neuraxial placement, increased from 35.1 ± 9.4 min to 50.6 ± 15.9 min (*p* < 0.0001), while total procedure time increased from 57.7 ± 24.3 to 76.8 ± 27.4 min (*p* < 0.0001). EBL was significantly higher in the superobese patients: 902 ± 294 mL versus normal BMI patients: 712 ± 230 mL (p < 0.0001). 1-min Apgar scores declined with higher BMI, but 5-min scores were comparable across groups.

**Conclusions:**

Superobesity is associated with specific intraoperative negative outcomes during cesarean delivery under neuraxial anesthesia. Our study findings highlight the need for anticipatory anesthetic planning and tailored hemodynamic management strategies in high-BMI parturients to optimize maternal and neonatal outcomes.

**Supplementary Information:**

The online version contains supplementary material available at 10.1007/s00404-026-08408-0.

## What does this study add to the clinical work

**Table:** 

Super-obese parturients represent a high-risk obstetric population with greater hemodynamic instability, prolonged surgical duration, and significant technical challenges during emergent cesarean delivery, potentially impacting fetal outcomes. This underscores the need for proactive multidisciplinary planning, early neuraxial optimization, vigilant management of hypotension, timely surgical preparedness, and comprehensive patient counseling to optimize maternal and fetal outcomes.	

## Introduction

Obesity remains a significant public health concern in the United States, with profound implications for maternal and perinatal outcomes. According to the National Health Statistics Reports from 2017 to 2020, the prevalence of obesity, defined as body mass index (BMI) ≥ 30 kg/m^2^ among reproductive-age women, approached 40%, while severe obesity (BMI ≥ 40 kg/m^2^) affected 12.4% of this population [1]. The presence of obesity during pregnancy is associated with an increased rate of elective or emergent cesarean delivery. In a cohort study of 438 singleton pregnancies, among parturients with class III obesity undergoing labor induction, 46% required cesarean delivery, and this rate rose to 63% in parturients with a BMI of 50–60 kg/m^2^ [2].

Obese parturients undergoing cesarean delivery present distinct anesthetic challenges, including technically difficult intravenous access, monitoring, airway management, and neuraxial placement, as well as an elevated risk of failed conversion from labor epidural to surgical anesthesia [3, 4]. Morbidly obese parturients also exhibit higher rates of maternal hypotension and prolonged fetal heart rate decelerations following labor epidural analgesia [5]. Benevides et al. identified a significant association between morbid obesity (BMI ≥ 40 kg/m^2^) and increased incidence of hypotension and fetal acidosis with neuraxial anesthesia for cesarean delivery [6–8].

Although these studies support that obesity exacerbates hypotension following neuraxial anesthesia, they lack detailed analyses of vasopressor use and hemodynamic management strategies. Moreover, limited data exist regarding anesthetic and surgical outcomes in the superobese obstetric population (BMI ≥ 50 kg/m^2^), despite its growing prevalence. In this study, we aimed to further stratify and quantify the degree of hypotension and vasopressor requirements in superobese parturients following neuraxial anesthesia. Additionally, we examined the association of superobesity with prolonged surgical duration, increased estimated blood loss, and neonatal outcomes.

We hypothesized that superobesity is associated with increased hemodynamic instability, higher vasopressor requirements, prolonged surgical duration, greater intraoperative blood loss during cesarean delivery, and lower Apgar scores.

## Methods

This retrospective cohort study was conducted at the University of Alabama at Birmingham following approval from its Institutional Review Board (IRB-300013075). Parturients who underwent cesarean delivery under neuraxial anesthesia between January 1, 2018, and December 31, 2023, were included in the study. Patients were excluded if they had a documented history of cardiac disease during pregnancy (e.g., peripartum cardiomyopathy, valvular disorders, arrhythmias, or congenital heart disease), failed neuraxial anesthesia or underwent cesarean hysterectomy for placenta accreta spectrum. A total of 2,051 charts were reviewed, of which 1,996 parturients met the inclusion criteria. These were stratified into six body mass index (BMI) categories according to the World Health Organization definitions: normal weight (BMI 18.5–24.9) (*N* = 116), overweight (BMI 25.0–29.9) (*N* = 430), obese class I (BMI 30.0–34.9) (*N* = 516), obese class II (BMI 35.0–39.9) (*N* = 416), obese class III (BMI ≥ 40.0) (*N* = 378), and obese class IV (BMI ≥ 50.0) (*N* = 140). Superobese parturients, corresponding to obesity class IV, are defined as those with a body mass index (BMI) ≥ 50 kg/m^2^.

At our institution, neuraxial anesthesia for cesarean delivery is provided using spinal, combined spinal–epidural (CSE), or epidural techniques, selected based on patient characteristics and obstetric indications. Spinal anesthesia is used for primary cesarean deliveries, while CSE is preferred for repeat cesarean deliveries with anticipated longer durations. Primary epidural anesthesia is the neuraxial anesthetic plan when patients laboring on the floor with epidurals in situ for labor analgesia undergo cesarean delivery. Spinal anesthesia is administered as a single intrathecal injection of 12 mg hyperbaric bupivacaine 0.75%, 100 mcg preservative-free morphine, and 15 mcg fentanyl. The combined spinal–epidural (CSE) technique involves intrathecal injection of 12 mg isobaric bupivacaine with 100 mcg preservative-free morphine, and 15 mcg fentanyl, followed by epidural catheter placement. All the neuraxial procedures are aimed to achieve a sensory block to the T4 dermatome. Following intrathecal administration, a prophylactic phenylephrine infusion is initiated at 0.5 µg/kg/min based on actual body weight and titrated to maintain maternal mean arterial pressure within ± 20% of baseline. In patients with preexisting labor epidurals, 2% lidocaine with epinephrine is administered to achieve a surgical sensory level to T4, and the phenylephrine infusion is initiated at the time of supplemental local anesthetic administration to establish surgical anesthesia. For all cesarean deliveries, systolic, diastolic, and mean arterial blood pressures are measured noninvasively at 3-min intervals throughout the duration of the surgical procedure.

The primary outcome of the study was the incidence and severity of intraoperative hypotension, defined as a decline in mean arterial pressure of ≥ 20% from baseline. This was assessed using serial noninvasive blood pressure (NIBP) measurements obtained at 3-min intervals for the first 30 min following neuraxial anesthesia placement. Clinical covariates included the presence of maternal gestational diabetes, primary (essential) arterial hypertension, and hypertensive disorders of pregnancy (preeclampsia and eclampsia). Further hypotension was stratified by the neuraxial technique employed. Secondary outcomes included (1) duration of neuraxial anesthesia placement (including time for patient positioning, confirming IV access); (2) elapsed time from surgical start to fetal delivery; (3) total surgical duration; (4) estimated blood loss (EBL); and (5) Apgar scores at 1 and 5 min.

### Statistical analysis

We summarized all continuous outcome measures by BMI category using means and standard deviations. To assess differences in the decrease in NIBP during the first 30 min following neuraxial anesthesia, we compared NIBP values across BMI groups using one-way analysis of variance (ANOVA). Multivariate regression analysis was performed to evaluate associations between maternal characteristics and perioperative outcomes. A multivariable linear mixed-effects model (LMM) was used to assess changes in blood pressure over time. For each patient, NIBP measurements were recorded every 3-min intervals during the first 30 min of the procedure, resulting in 10 time points per individual. This allowed the blood pressure trajectories over time to be assessed in relation to BMI. Following established modeling techniques (Singer & Willett, 2003), we incorporated a stepwise approach to account for the known physiological pattern of a blood pressure drop during the first 15 min of neuraxial anesthesia, followed by stabilization. Our model specifically examined whether BMI was associated with

1. Baseline NIBP
2. Rate of change in NIBP over time
3. The shift in blood pressure trend after the initial 15 min.

To improve interpretability, BMI was treated as a continuous variable and centered at the sample mean. This ensured that the intercept and slope of the model corresponded to individuals with an average BMI. The initial analyses indicated a relatively linear relationship between BMI and blood pressure, supporting this approach.

Random intercepts and slopes were included to account for individual-level differences in baseline blood pressure and its rate of change over time. Covariates including preexisting primary (essential) arterial hypertension, preeclampsia, and diabetes were adjusted for in the model. Estimates from the model are displayed graphically and are calculated using predictive margins, which calculate average predicted outcomes from the fitted regression model by averaging predictions across the observed data.

Differences in procedure time, Apgar, estimated blood loss, demographics, and patient characteristics by BMI categories are examined using one-way ANOVA or Kruskal–Wallis tests for continuous variables, and Chi-squared tests for categorical variables. For phenylephrine analyses, log linear regression is used to account for the skewed distribution.

Since this was a retrospective study, some data were missing. The missingness was confined to the outcomes; covariates were complete, and outcome missingness was modest (≤ 6%); therefore, multiple imputations were deemed unnecessary. Cross-sectional models were used for outcome-specific complete-case analyses, while the longitudinal outcome was analyzed using maximum-likelihood linear mixed models that retain partially observed outcomes [9, 10].

## Results

**Fig. 1:**
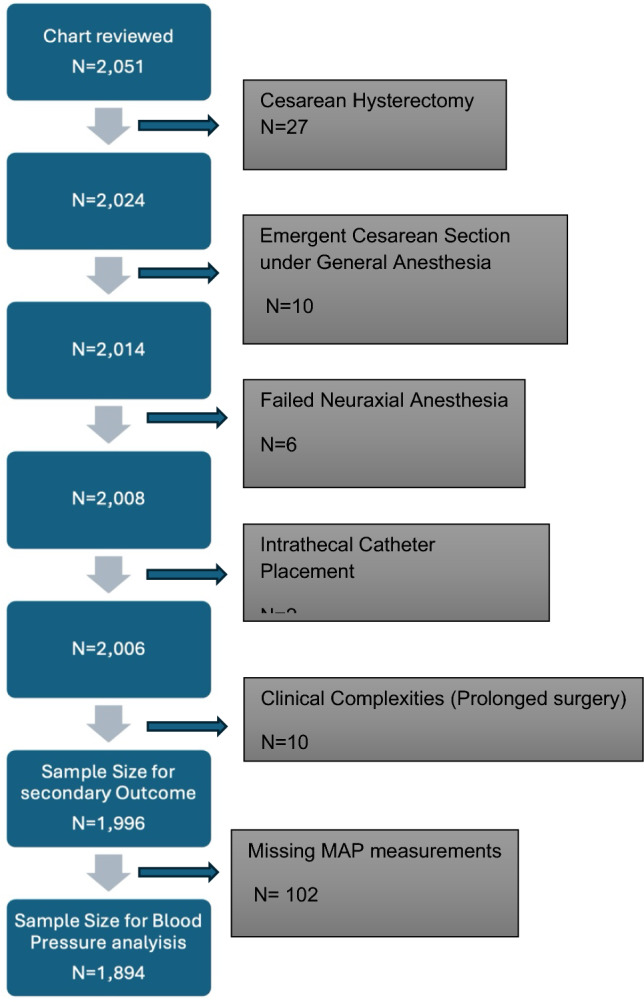
STROBE diagram of study population. This flow diagram outlines the selection process of the study cohort for a retrospective analysis of cesarean deliveries performed under neuraxial anesthesia

Of the 2051 cesarean delivery charts reviewed, 1996 patients were included in the study. 55 were excluded due to predefined criteria (Fig. 1): cesarean hysterectomy for placenta accreta spectrum (*n* = 27), emergent cesarean under general anesthesia (*n* = 10), failed neuraxial anesthesia (*n* = 6), intrathecal catheter placements (*n* = 2), and prolonged surgical duration due to clinical complexities (*n* = 10), including classical cesarean incisions, concurrent urologic interventions, ex utero intrapartum treatment (EXIT) procedures, and one uterine transplant case. Blood pressure management for 102 patients could not be extracted, so the sample size for the primary outcome was reduced to 1894.

Demographic data (Table 1) showed the proportion of White parturients was 43.1% in the normal weight group and 25.7% in the superobese group, whereas the proportion of African American parturients was 32.8% in the normal weight group and 59.3% in the superobese group (*p* < 0.001). Maternal age, gestational age at delivery, and parity were similar across BMI categories (*p* > 0.05). Clinical comorbidities increased substantially with BMI: gestational diabetes rose from 6.9% to 32.1%, gestational hypertension from 9.5% to 40.7%, and preeclampsia from 8.6% to 25.7% (all *p* < 0.001). The use of CSE increased with BMI, comprising 67.9% of cases in the superobese group compared to 61.2% use of spinal anesthesia in the normal weight cohort.

**Table 1:** Demographic data

	Total (*n* = 1996)	Normal weight (*n* = 116)	Overweight (*n* = 430)	Obese I (*n* = 516)	Obese II (*n* = 416)	Obese III (*n* = 378)	Superobese (*n* = 140)	*P*-value
	Mean	Mean	Mean	Mean	Mean	Mean	Mean	
BMI	35.90 (9.19)	23.12 (1.51)	27.64 (1.39)	32.40 (1.46)	37.29 (1.45)	44.04 (2.83)	58.61 (8.36)	–
Maternal age	30.63 (5.55)	29.55 (5.90)	31.00 (5.78)	30.78 (5.71)	30.72 (5.29)	30.43 (5.39)	30.09 (5.10)	0.13
Race								
White	34.4%	43.1%	41.6%	36.4%	30.8%	27.8%	25.7%	< 0.0001
Black	39.0%	32.8%	28.4%	32.6%	43.5%	49.2%	59.3%	
Other	12.6%	10.3%	16.7%	13.2%	10.1%	11.4%	10.7%	
Hispanic	14.0%	13.8%	13.3%	17.8%	15.6%	11.6%	4.3%	
Gestational age	37.91 (2.63)	37.56 (2.00)	37.97 (2.04)	38.00 (1.96)	38.02 (1.96)	37.90 (4.39)	37.33 (1.96)	0.07
Parity	2.48 (1.26)	2.48 (1.42)	2.45 (1.21)	2.50 (1.26)	2.52 (1.28)	2.50 (1.27)	2.33 (1.21)	0.73
Parity category								
0	2.1%	1.7%	2.1%	3.1%	1.0%	1.6%	3.6%	0.432
1	18.3%	22.4%	16.5%	16.5%	20.2%	18.8%	20.7%	
2	36.2%	37.1%	39.3%	35.1%	33.7%	36.8%	36.4%	
3 or more	43.3%	38.8%	42.1%	45.3%	45.2%	42.9%	39.3%	
Gestational diabetes	17.1%	6.9%	9.3%	14.1%	19.7%	24.6%	32.1%	< 0.0001
Gestational hypertension	16.8%	9.5%	8.6%	11.8%	15.1%	28.3%	40.7%	< 0.0001
Preeclampsia	13.3%	8.6%	9.3%	11.6%	13.0%	17.2%	25.7%	< 0.0001
Primary technique								
Epidural	7.1%	7.8%	4.9%	6.0%	5.3%	9.0%	17.9%	< 0.0001
CSE	39.0%	31.0%	31.4%	32.4%	41.3%	46.0%	67.9%	
Spinal	53.9%	61.2%	63.7%	61.6%	53.4%	45.0%	14.3%	
Type of procedure								
Primary not BTL	47.1%	44.8%	48.1%	49.2%	45.9%	42.3%	54.3%	0.121
Primary BTL	6.0%	2.6%	4.2%	7.0%	7.5%	5.8%	6.4%	
Repeat not BTL	32.0%	36.2%	34.4%	30.4%	31.7%	32.5%	26.4%	
Repeat BTL	14.9%	16.4%	13.3%	13.4%	14.9%	19.3%	12.9%	

Maternal characteristics across six BMI categories showed significant racial variation with fewer White and more Black patients at higher BMIs (*p* < 0.001), and increased rates of gestational diabetes, chronic hypertension, and preeclampsia with rising BMI (all *p* < 0.001). Significant tests indicate significant associations by weight status obtained from ANOVA for continuous variables and *χ*^2^ for categorical variables

**Fig. 2:**
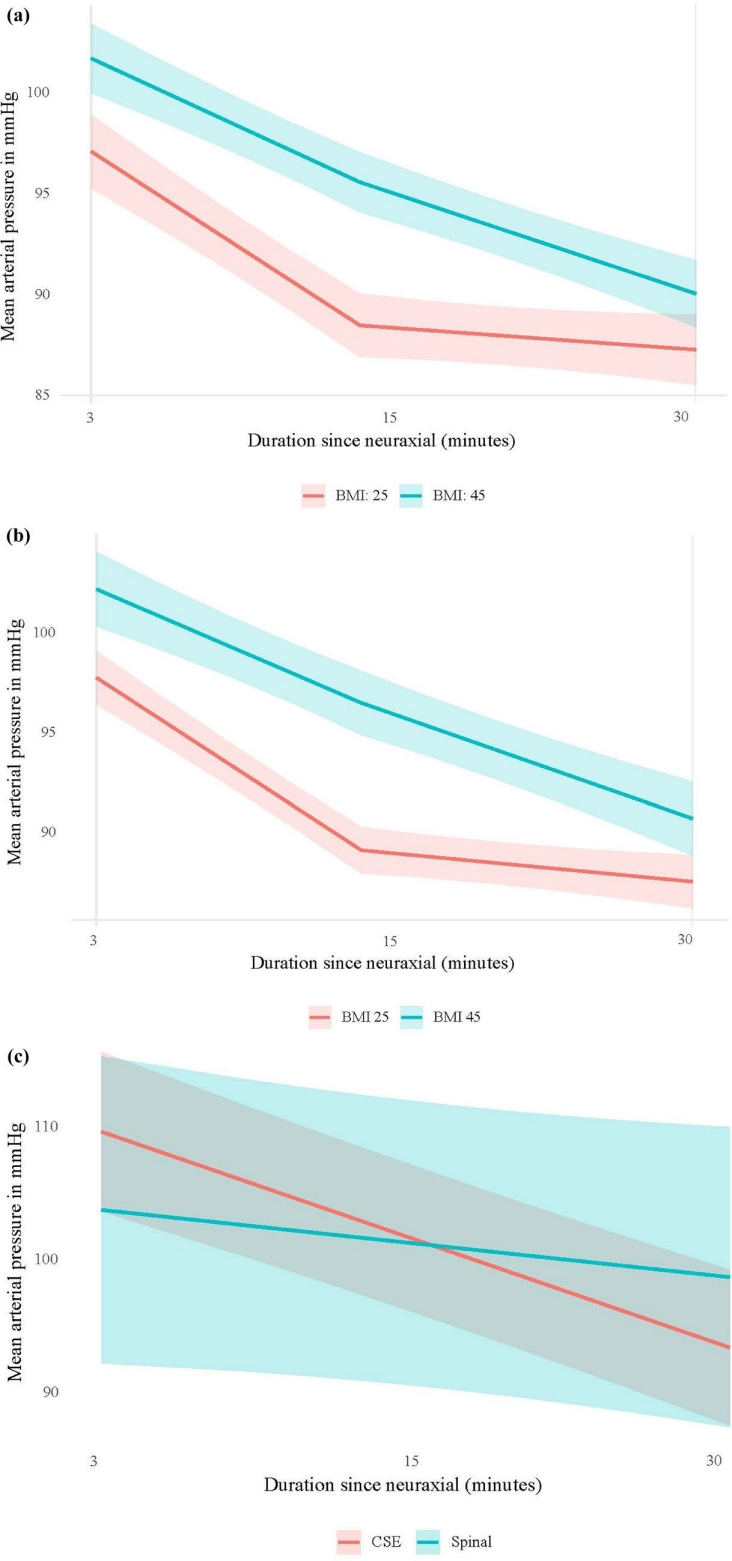
**a** Mean arterial pressure trends following combined spinal anesthesia (CSE) by BMI category. This figure illustrates the trajectory of mean arterial pressure (MAP) over 30 min following combined spinal anesthesia (CSE) in parturient stratified BMI 25 and 45 which are mean + SD in normal weight and obese groups. **b** Mean arterial pressure trends following spinal anesthesia by body mass index. This figure illustrates changes in mean arterial pressure (MAP) over a 30-min period following spinal anesthesia, stratified by body mass index (BMI) 25 and 45 taken as mean + SD for normal weight and obese parturient. **c** Mean arterial pressure trends following spinal and CSE within the superobese group. This figure illustrates changes in mean arterial pressure (MAP) over a 30-min period following spinal anesthesia and CSE within the superobese group

The relationship between decrease in MAP and BMI was analyzed using BMI as a continuous variable to enhance the statistical power and precision of the analysis, rather than categorizing BMI into discrete groups. When modeled continuously, MAP demonstrated a biphasic decline following neuraxial anesthesia, characterized by an initial steep decrease during the first 15 min postblockade, followed by a more gradual decline thereafter. This biphasic pattern was consistently observed across both the combined spinal-epidural (CSE) and spinal anesthesia cohorts; however, the magnitude and slope of MAP decline varied as a function of BMI, indicating that higher BMI was associated with a slower initial decline but a more sustained hypotensive response over time. In the CSE and spinal groups, for every one-unit increase in BMI, there was a 0.03 mmHg less decline during the first 15 min and a 0.07 mmHg greater decline during the last 15 min. This association is displayed graphically in Fig. 2a and b for individuals with a mean BMI of 25 (mean + 1 SD for normal weight) and a BMI of 45 (mean + 1 SD for obese parturients). In the epidural group, there were no significant differences in blood pressure trajectories. Among this group, MAP decreased steadily during the 30 min, and blood pressure trajectories (both beginning value and change overtime) did not vary by BMI. These observations indicate a delayed but more pronounced hypotensive response in the superobese population after CSE and spinal anesthesia. Further stratification of MAP response by neuraxial technique within the superobese group (Fig. 2c) demonstrated that patients receiving spinal anesthesia had a nonsignificant decline in MAP over 30 min (− 0.56, *p* = 0.21), whereas those receiving CSE experienced a significant decrease (− 1.81, *p* < 0.001). In addition, the overall change over time differed significantly between the CSE and spinal groups (*p* = 0.016). Despite this difference in trajectories, there were no statistically significant differences in marginal mean MAP between the two groups at any observed time point, as reflected by overlapping confidence intervals in Fig. 2c.

**Fig. 3:**
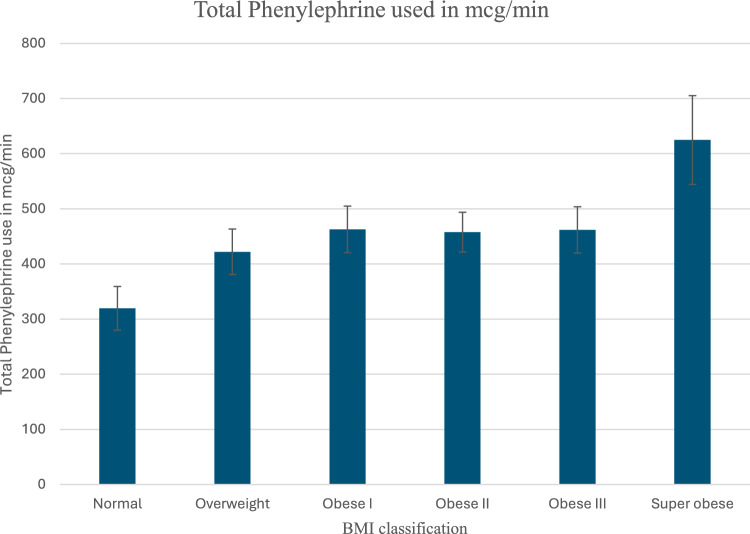
Phenylephrine infusion rates by obesity class. Total phenylephrine infusion rate (in micrograms per minute) administered to maintain hemodynamic stability following neuraxial anesthesia, stratified by maternal body mass index (BMI) categories. A 1 unit increase in BMI was associated with a 1.61% increase in phenylephrine use in the adjusted models for comorbidities including hypertensive disease of pregnancy and gestational diabetes

In our clinical practice, phenylephrine is administered as a continuous infusion dosed in micrograms per kilogram per minute (mcg/kg/min) based on actual body weight. The delayed hypotensive response observed in patients with higher BMI may, in part, be attributable to the administration of higher absolute doses of phenylephrine in this group as it is programmed in the pump with actual body weight. Although phenylephrine administration data in real time could not be extracted, total intraoperative phenylephrine usage was significantly higher in individuals with elevated BMI as it was dosed by actual body weight (Fig. 3). Despite the higher administered phenylephrine dose, these individuals had persistently decreasing blood pressure. This indicated that in individuals with higher BMI, hypotension was refractory to increased phenylephrine doses. A second-line vasopressor—most commonly ephedrine—was typically administered in patients who developed reflex bradycardia (HR < 60/min) during a phenylephrine infusion. Ephedrine is given as an IV bolus dose of 5–10 mg. When ephedrine utilization was analyzed across BMI categories, there were no statistically significant differences (Supplemental Table 1) in the total dose received among groups (*p* = 0.0849),

A linear mixed-effects model was used to study the effect of multiple factors on MAP including BMI, time, total phenylephrine use (supplemental Table 3a and b). Adjustment for phenylephrine dose did not materially alter the relationship between BMI and decline in MAP over time and phenylephrine was not independently associated with MAP (*p* = 0.58). Additionally, obstetric comorbidities including hypertension, preeclampsia, and gestational diabetes were not significant predictors in the adjusted model (supplemental Table 3a and b).

**Fig. 4:**
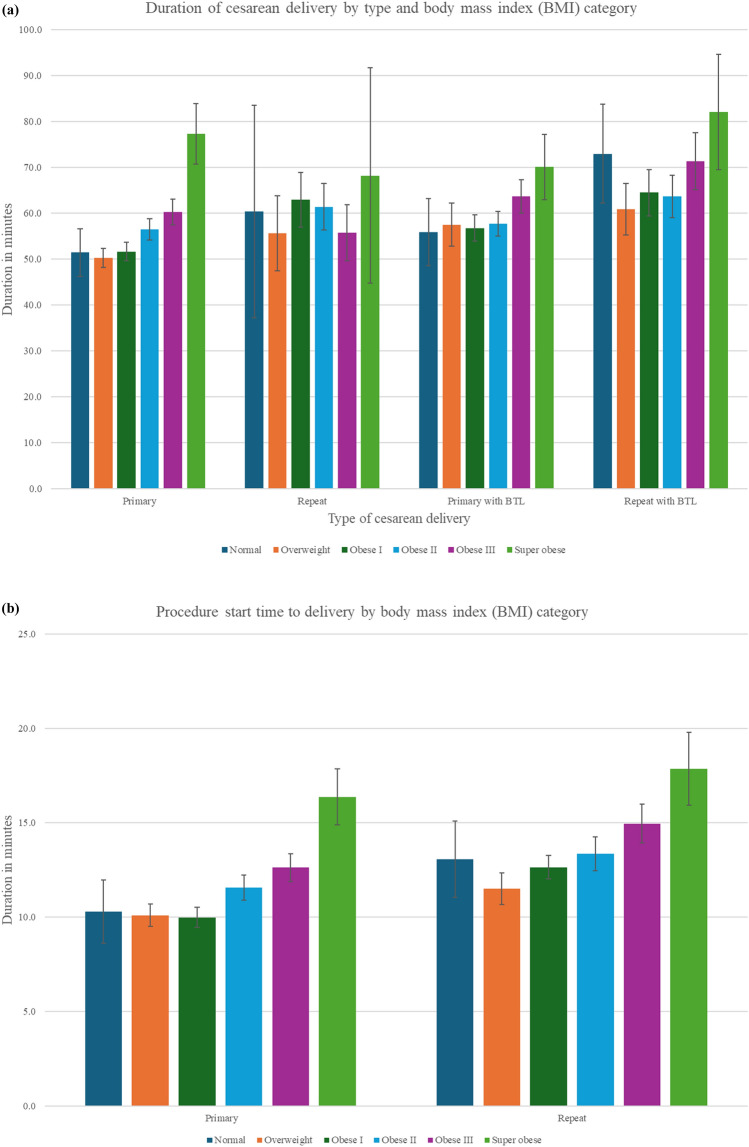

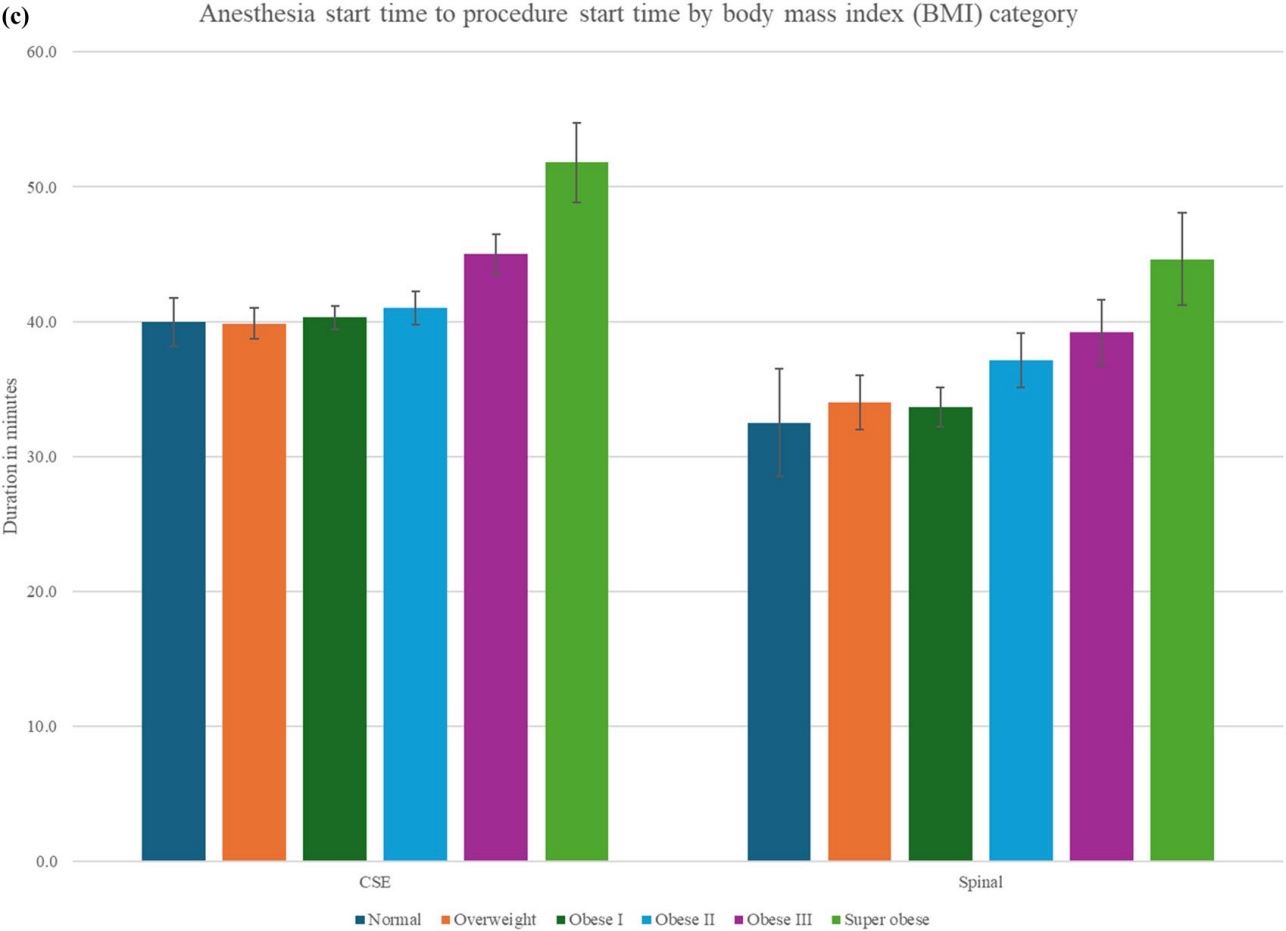
**a **Duration of cesarean delivery by type and body mass index (BMI) category. The bar graph displays the mean operative time (in minutes) for four types of cesarean deliveries—primary, repeat, primary with bilateral tubal ligation (BTL), and repeat cesarean delivery with BTL—stratified by BMI. Error bars represent standard deviation. *BTL* bilateral tubal ligation; *BMI* body mass index. **b** Procedure start to delivery time. This illustrates the mean procedure time to delivery (minutes) with standard error bars, stratified by BMI category and by type of cesarean delivery (primary vs. repeat). Across all cesarean delivery types, increasing BMI is associated with longer times from procedure start-to-uterine incision. **c** Anesthesia start time to procedure start time. Bar graph showing mean duration (minutes) from initiation of anesthesia to surgical start, with standard error bars, stratified by BMI category and by type of neuraxial technique (combined spinal–epidural [CSE] vs. spinal). Among all patients receiving CSE or spinal, the time on anesthesia prior to incision increased progressively with BMI

Mean total operative time (Fig. 4a, supplemental Table 2) increased with BMI, from 57.7 ± 24.3 min to 76.8 ± 27.4 min across BMI groups (*p* < 0.0001). On further subanalysis based on the type of cesarean deliveries, in primary cesarean deliveries, durations increased from 53.5 ± 23.6 to 78.1 ± 28.5 min, and in primary cesarean with bilateral tubal ligations (BTL) from 55.7 ± 17.7 in the overweight group to 70.0 ± 31.8 min in the superobese (*p* < 0.01). Repeat cesareans with BTL in superobese patients had a mean duration of 82.1 ± 26.9 min. The interval from surgical procedure start to fetal delivery (Fig. 4b, supplemental Table 1) increased progressively with higher BMI mean of 11.8 ± 9.1 min in normal weight versus 17.3 ± 9.3 min in superobese (*p* < 0.0001). This increased trend persisted in both primary cesarean deliveries (10.3 ± 7.5 min to 16.5 ± 9.0 min) and repeat cesarean deliveries (13.1 ± 0.3 min to 18.7 ± 9.4 min). The time from patient arrival to the operating room (anesthesia start time) to surgical start increased significantly across BMI categories from 35.1 ± 9.4 min to 50.6 ± 15.9 min (*p* < 0.0001). With CSE, this duration rose to 51.8 ± 1.6 min; with spinal anesthesia, it reached 44.6 ± 9.2 min in the superobese group (Fig. 4c, supplement Table 2). This duration includes patient positioning, IV access assessment and replacement if needed, and neuraxial placement.

**Fig. 5:**
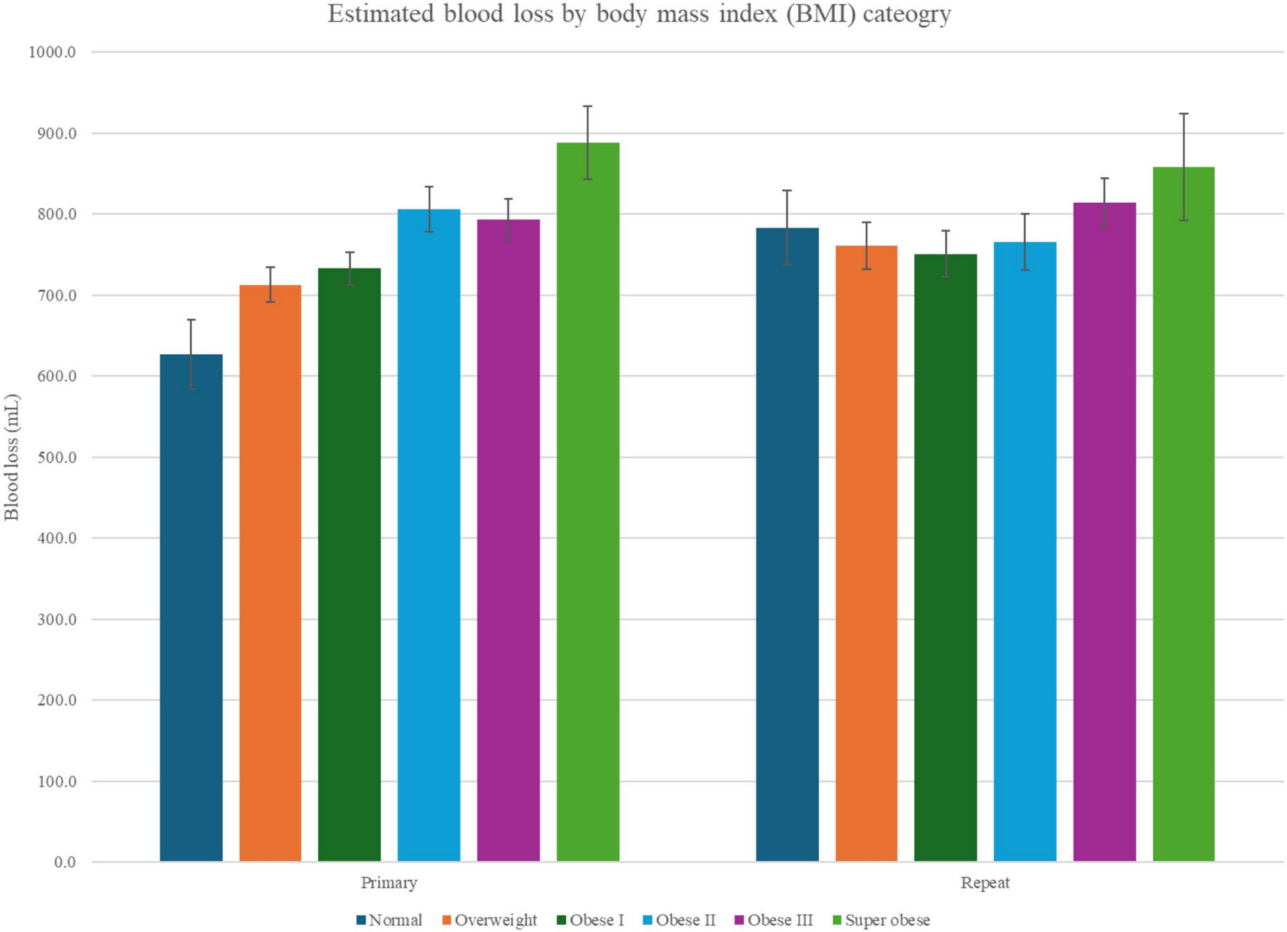
Estimated blood loss. Bar graph depicting mean estimated blood loss (EBL), in mL, with standard error bars, stratified by BMI category (normal weight, overweight, obese I, obese II, obese III, and superobese) and by type of cesarean delivery (primary versus repeat). Among all cesarean deliveries, EBL increased with higher maternal BMI, regardless of primary or repeat surgical status

EBL showed a positive association with BMI. EBL was significantly higher in superobese patients (902 ± 29.4 ml) compared to normal weight (712 ± 230 ml), *p* < 0.0001). Repeat cesarean deliveries were consistently associated with greater EBL values across BMI strata (Fig. 5). One-minute Apgar scores (supplemental Table 2a) declined with increasing BMI (7.38 ± 1.61 vs. 6.91 ± 1.96; *p* = 0.003, while 5-min Apgar scores remained stable across groups (*p* = 0.62) indicating transient neonatal depression in higher BMI patients. Differences remained significant after accounting for gestational age.

## Discussion

Our findings indicate that superobesity is associated with a sustained and progressive hypotensive response following neuraxial anesthesia. In patients with a mean BMI of 45 undergoing combined spinal–epidural (CSE) or spinal anesthesia, mean arterial pressure (MAP) exhibited a slower initial decline during the first 15 min following neuraxial placement; however, the decline persisted beyond 15 min despite ongoing phenylephrine administration. In contrast, individuals with a lower BMI exhibited a more pronounced drop in MAP within the first 15 min, followed by stabilization. thereafter, suggesting a more effective hemodynamic response to phenylephrine in this group. The attenuated initial MAP decline observed in the superobese cohort during the first 15 min may be attributable to higher phenylephrine dosing, administered in mcg/kg/min using actual body weight. Nonetheless, persistent MAP reduction beyond 15 min in the superobese group, despite higher vasopressor use, likely reflects altered physiology (reduced vascular tone, increased venous capacitance, aortocaval compression, and impaired sympathetic response) leading to a sustained vasodilatory state rather than a rebound effect from phenylephrine.

These findings expand upon prior literature associating maternal obesity with increased intraoperative hypotension and hemodynamic instability under neuraxial anesthesia as well as potential fetal compromise [5, 8]. Gaiser and Taylor et al. emphasized concealed aortocaval compression, prolonged operative duration, and altered pharmacodynamics in this high-risk group [4, 11]. Our study not only reinforces these associations but also provides quantitative estimates of blood pressure decline and vasopressor demand relative to BMI. The inclusion of a large sample size, particularly in the superobese group, strengthens the internal validity and generalizability of these results and highlights the need for individualized, BMI-tailored hemodynamic management protocols.

Furthermore, within the superobese cohort, the incidence of hypotension was higher with the CSE technique compared with spinal anesthesia alone. Although both techniques utilized 12 mg of intrathecal bupivacaine, the differential baricity between hyperbaric bupivacaine for spinal anesthesia and isobaric bupivacaine for CSE may have contributed to the observed hemodynamic differences. Prior literature suggests that intrathecal hyperbaric bupivacaine is associated with greater hemodynamic stability compared with isobaric bupivacaine [12]. At our academic institution, isobaric bupivacaine is preferred for CSE to facilitate epidural catheter placement while minimizing the risk of an inadequate block associated with prolonged sitting after hyperbaric bupivacaine due to its position-dependent intrathecal spread.

Operative times were significantly prolonged in the enrolled superobese group, with a 35% increase in total surgical duration and a 51% increase in skin-to-uterine incision time compared to those patients with normal BMI. These findings align with prior reports identifying technical challenges related to surgical exposure and intraoperative tissue handling in morbidly obese parturient [13]. Such delays may be especially consequential in emergent deliveries, where extended incision-to-delivery intervals could adversely affect fetal outcomes [14]. Proactive OR scheduling and case prioritization, according to fetal status and allocating extended operative time to accommodate anticipated delays, are essential in high-BMI patients requiring urgent or emergent intervention.

Additionally, the time from patient’s entry into the operating room to the surgical start was approximately 30% longer in the superobese group. This is likely attributable to anesthesia-related challenges, including difficult intravenous access, positioning difficulties, and complex neuraxial placement. Prior studies have supported the use of ultrasound to facilitate neuraxial placement in parturients with BMI > 44 [11, 15], an approach that may improve efficiency and success rates in this population.

Estimated blood loss during cesarean delivery was 25% to 40% greater in the superobese group, consistent with increased surgical complexity, duration and reduced uterine contractility previously described in this parturient cohort [16]. These findings provide obstetric and anesthesia providers with practical insight into the magnitude of additional blood loss expected in superobese parturients and further support anticipatory hemorrhage planning, including early availability of uterotonic agents and blood products.

Neonatal outcomes included lower 1-min Apgar scores in higher BMI groups, suggesting transient neonatal depression likely related to prolonged skin incision-to-delivery time, maternal hypotension, or uteroplacental insufficiency. The normalization of 5-min Apgar scores, however, is reassuring and indicative of effective neonatal resuscitation and vigilant intraoperative and peripartum maternal–fetal care at our institute. Prolonged surgery start-to-uterine incision time has been associated with lower Apgar scores [14]. To optimize fetal outcomes, prompt treatment of maternal hypotension and proactive planning for delivery in the setting of nonreassuring fetal heart tones are essential. This is particularly important given the potential delays associated with achieving neuraxial anesthesia and the prolonged interval from surgical start-to-uterine incision as determined above.

We underscore several critical anesthetic considerations for the management of superobese parturients. The use of ultrasound guidance for neuraxial blockade—whether for labor analgesia or surgical anesthesia—is strongly recommended, as it improves first-attempt success rates, reduces the number of needle redirections, and decreases procedural time and neuraxial failure rates [15, 17]. Early placement of labor epidurals can be advantageous by minimizing the risk of emergent airway management in the setting of difficult intubation and decreasing the decision-to-delivery time in parturients with deteriorating fetal status [18, 19]. However, these epidurals must be routinely assessed for efficacy, as failed conversions from labor analgesia to surgical anesthesia remain common in this population [21]. When selecting a neuraxial technique in the operating room for scheduled cesarean deliveries, clinicians must weigh the risk of high spinal block, increased hemodynamic lability, and prolonged surgical duration [8]. Although epidural-only techniques may reduce the incidence of hypotension, they are often slower to establish and may provide insufficient surgical anesthesia. Current evidence supports the use of CSE techniques in obese parturients, particularly in those with pregnancy-induced hypertension, due to the rapid onset of spinal anesthesia coupled with the flexibility to extend the block via the epidural catheter and avoiding potential airway complications in case of conversion of general anesthesia [22, 23].

To mitigate sustained hypotension, vigilant intraoperative monitoring and titration of vasopressors are essential. In addition to phenylephrine, second-line agents such as ephedrine or norepinephrine may be required. Timely obstetric decision-making is paramount in cases of evolving fetal distress, as delays can further prolong uterine incision-to-delivery intervals, potentially leading to fetal compromise [19]. Readiness to manage postpartum hemorrhage due to increased surgical blood loss and uterine atony is also vital. Effective perioperative planning for anticipated prolonged cesarean deliveries in this population must involve multidisciplinary coordination among anesthesiology, obstetric, and surgical teams. This is especially critical in patients with BMI > 50, in whom the maternal and neonatal risks are significantly heightened [3, 24]. Additionally, anesthesiologist should be cognizant of the higher prevalence of obesity among African American parturients, mirroring national trends in BMI distribution by race [20] and its correlation with increased rates of gestational comorbidities, including hypertensive disorders and gestational diabetes, which was demonstrated in our study in alignment with prior large-scale studies [25, 26]. Obesity disproportionately affects populations with lower income, limited educational attainment, and residence in under-resourced neighborhoods [27]. In obstetric populations, these structural inequities intersect with pregnancy-related vulnerability, contributing to disproportionate risks of maternal morbidity and mortality. Addressing these disparities requires all health care providers to deliver stigma-free, equitable care; implement standardized, evidence-based, risk-driven clinical protocols; and ensure early, multidisciplinary prenatal engagement for pregnant patients with obesity.

Limitations to the current study must be acknowledged. As a retrospective study, the analysis is subject to limitations in data granularity and potential misclassification. In particular, we were unable to capture time-resolved data on phenylephrine infusions, limiting our ability to assess real-time vasopressor responsiveness. Additionally, there is an institutional-based bias of dosing phenylephrine according to actual body weight masking the initial hypotensive response in superobese and morbidly obese parturient. From a theoretical pharmacologic perspective, vasopressors are hydrophilic agents with a small volume of distribution, which supports dosing based on IBW [28]. However, existing studies in critically ill ICU populations have demonstrated no clinically significant differences between weight-based and non-weight-based vasopressor dosing strategies in terms of hemodynamic outcomes [29]. Given the limitations of the current evidence base, this represents an important area for further investigation, and we recommend a future prospective study comparing IBW-based, ABW-based, and fixed-dose phenylephrine infusion strategies for the management of neuraxial anesthesia associated hypotension in this population. Additionally, given the greater hypotensive response observed among superobese parturients receiving combined spinal–epidural (CSE) anesthesia, further studies are warranted to determine whether reducing the initial intrathecal dose of isobaric bupivacaine and/or substituting hyperbaric bupivacaine for CSE may attenuate hemodynamic perturbations in this population. Future investigations should also incorporate objective neonatal outcome measures, including umbilical cord blood gas analysis, which were not available for extraction in the present study.

## Conclusion

Increasing maternal BMI is associated with a higher prevalence of maternal comorbidities, greater incidence of intraoperative hypotension, increased vasopressor requirements, prolonged operative durations, elevated blood loss, and transient neonatal depression. These findings highlight the importance of individualized anesthetic planning and proactive perioperative strategies for obese parturient. Risk-specific counseling should be provided to patients, and multidisciplinary teams should anticipate longer surgical times and greater blood loss, ensuring appropriate preparation and resource allocation for optimal maternal and neonatal outcomes.

## Supplementary Information

Below is the link to the electronic supplementary material.

Ephedrine usage. This table summarized ephedrine usage (mg) over different BMI groups. Supplementary file1 (DOCX 14 KB)

Secondary outcomes/Cesarean section outcomes. This table summarizes operative times, anesthesia duration, neonatal Apgar scores, and estimated blood loss across BMI categories from normal weight to superobese. Increasing BMI was associated with longer procedure times to delivery, greater time under anesthesia before incision, and longer total operative duration and Estimated blood loss. Supplementary file2 (DOCX 20 KB)

a Mixed effect model for patients with CSE Mixed-effects linear regression model evaluating the association between time (wave), body mass index (BMI, centered), and hemodynamic response among patients receiving CSE. Coefficients are presented with 95% confidence intervals and corresponding p-values. b Mixed effect Model for patients with Spinal Anesthesia Mixed-effects linear regression model evaluating the association between time (wave), body mass index (BMI, centered), and hemodynamic response among patients receiving spinal anesthesia. Coefficients are presented with 95% confidence intervals and corresponding p-values. Supplementary file3 (DOCX 19 KB)

## Data Availability

No datasets were generated or analyzed during the current study.
