# Correction: Intraoperative hemodynamics and anesthetic implications in superobese parturients undergoing cesarean delivery: a retrospective cohort analysis

**DOI:** 10.1007/s00404-026-08502-3

**Published:** 2026-07-02

**Authors:** Teshi Kaushik, Andrew Hackney, Ayesha Bryant, Elizabeth Baker, Sijules Abongwa, Brant M. Wagener, Michael Arnold Frölich

**Affiliations:** Department of Anesthesiology and Perioperative Medicine, University of Alabama at Birmingham, Heersink School of Medicine, Birmingham, AL USA

**Correction: Archives of Gynecology and Obstetrics (2026) 313:152** 10.1007/s00404-026-08408-0

In this article, the author’s name Michael Arnold Frölich was incorrectly written as Michael Fröelich. The original article has been corrected.

