# Supplementary material for: Intraoperative hemodynamics and anesthetic implications in superobese parturients undergoing cesarean delivery: a retrospective cohort analysis
Source: Arch Gynecol Obstet. 2026 Apr 4;313(1):152. doi: 10.1007/s00404-026-08408-0 (PMC13050343; doi:10.1007/s00404-026-08408-0)
Supplement: Supplementary file 1 — Ephedrine usage. This table summarized ephedrine usage (mg) over different BMI groups. Supplementary file1 (DOCX 14 KB) [file 404_2026_8408_MOESM1_ESM.docx]

| **Ephedrine use** | | |
| --- | --- | --- |
|  | Total mcg Received | |
|  | Mean | Std Dev |
| Normal | 21.85 | 17.82 |
| Overweight | 25.68 | 21.48 |
| Obese 1 | 28.53 | 20.53 |
| Obese 2 | 26.26 | 19.99 |
| Obese 3 | 25.15 | 22.49 |
| Superobese | 30.19 | 19.72 |

**Supplemental Table 1 Ephedrine usage by BMI category.** This table summarizes the mean (± SD) total ephedrine dose received (mcg) across BMI groups.
