# Supplementary material for: Intraoperative hemodynamics and anesthetic implications in superobese parturients undergoing cesarean delivery: a retrospective cohort analysis
Source: Arch Gynecol Obstet. 2026 Apr 4;313(1):152. doi: 10.1007/s00404-026-08408-0 (PMC13050343; doi:10.1007/s00404-026-08408-0)
Supplement: Supplementary file 2 — Secondary outcomes/Cesarean section outcomes. This table summarizes operative times, anesthesia duration, neonatal Apgar scores, and estimated blood loss across BMI categories from normal weight to superobese. Increasing BMI was associated with longer procedure times to delivery, greater time under anesthesia before incision, and longer total operative duration and Estimated blood loss. Supplementary file2 (DOCX 20 KB) [file 404_2026_8408_MOESM2_ESM.docx]

| **Secondary outcomes/C-Section outcomes** | | | | | | |
| --- | --- | --- | --- | --- | --- | --- |
|  | Normal | Overweight | Obese I | Obese II | Obese III | Superobese |
| Skin incision to fetal delivery (n=1,994) | 11.8 (9.1) | 10.8 (6.1) | 11.2 (6.2) | 12.6 (7.2) | 13.9 (7.2)** | 17.3 (9.3)*** |
| Not specified  (n=51)^b^ | a | 10.1 (4.6) | 11.9 (6.2) | 12.7 (6.7) | a | a |
| Repeat (n=936) | 13.1 (10.3) | 11.6 (5.9) | 12.6 (6.8) | 13.7 (7.6) | 15.0 (7.2)+ | 18.7 (9.4)*** |
| Primary  (n=1,007) | 10.3 (7.5) | 10.1 (6.3) | 10.0 (5.4) | 11.5 (6.8) | 12.7 (6.9)* | 16.5 (9.0)*** |
| Time on anesthesia till procedure (n=1,852)^c^ | 35.1 (9.4) | 36.0 (11.4) | 36.2 (9.8) | 39.0 (12.7)** | 42.0 (14.0)*** | 50.6 (15.9)*** |
| CSE (n=777) | 40.2 (11.9) | 39.9 (11.6) | 40.7 (9.8) | 41.1 (13.4) | 44.8 (16.1)+ | 51.8 (16.8)*** |
| Spinal (n=1,075) | 32.5 (6.4) | 34.0 (10.8) | 33.8 (9.0) | 37.3 (11.9)* | 39.2 (10.7)*** | 44.6 (9.2)*** |
| Total procedure time (n=1,994) | 57.7 (24.3) | 55.3 (22.9) | 56.6 (20.9) | 58.9 (17.7) | 63.8 (21.6)** | 76.8 (27.4)*** |
| Primary  (n=939) | 53.5 (23.6) | 51.8 (17.2) | 53.4 (22.1) | 57.1 (18.2) | 60.9 (18.9)* | 78.1 (28.5)*** |
| Primary with  BLT (n=119)^b^ | a | 55.7 (17.7) | 62.9 (18.3) | 62.3 (14.2) | 55.8 (14.6) | 70.0 (31.8)+ |
| Repeat (n=638) | 55.9 (23.8) | 57.5 (29.2) | 56.7 (18.0) | 58.2 (16.5) | 64.6 (21.5)* | 73.2 (24.5)** |
| Repeat with BLT  (n=298) | 73.0 (23.7) | 62.1 (21.7)+ | 64.5 (21.3) | 64.4 (19.0) | 71.3 (26.8) | 82.1 (26.9) |
| APGAR 1 minute (n=1,879) | 7.5 (1.6) | 7.5 (1.5) | 7.5 (1.6) | 7.4 (1.5) | 7.2 (1.8)+ | 6.9 (2.0)** |
| APGAR 5 minute (n=1,876) | 8.7 (0.8) | 8.6 (1.0) | 8.6 (0.9) | 8.5 (0.9) | 8.6 (0.8) | 8.5 (0.8)+ |
| EBL (n=1,909) | 712 (230) | 742 (226) | 762 (345)+ | 792 (281)** | 811 (258)** | 902 (294)*** |
| Not specified  (n=50) | a | 851 (389) | 1431 (1460) | 987 (356) | a | 1700 (447) |
| Repeat (n=886) | 783 (253) | 765 (221) | 751 (208) | 769 (300) | 826 (301) | 863 (207)+ |
| Primary (n=973) | 627 (169) | 711 (209)* | 730 (190)** | 805 (258)*** | 793 (202)*** | 877 (264)*** |
| + p<0.01, * p<0.05, ** p<0.01, *** p<0.001 | | | | | | |
| Significant tests indicate significant differences between normal weight and that weight status using paired linear contrasts. | | | | | | |
| a Indicates cell size <5. | | | | | | |
| b Reference group is normal/overweight. | | | | | | |
| c Excludes epidural. | | | | | | |

**Supplemental Table 1 Secondary outcomes / Cesarean section outcomes** This table summarizes operative times, anesthesia duration, neonatal Apgar scores, and estimated blood loss across BMI categories from normal weight to superobese. Increasing BMI was associated with longer procedure times to delivery, greater time under anesthesia before incision, and longer total operative duration and Estimated blood loss.
