# Supplementary material for: Intraoperative hemodynamics and anesthetic implications in superobese parturients undergoing cesarean delivery: a retrospective cohort analysis
Source: Arch Gynecol Obstet. 2026 Apr 4;313(1):152. doi: 10.1007/s00404-026-08408-0 (PMC13050343; doi:10.1007/s00404-026-08408-0)
Supplement: Supplementary file 3 — a Mixed effect model for patients with CSE Mixed-effects linear regression model evaluating the association between time (wave), body mass index (BMI, centered), and hemodynamic response among patients receiving CSE. Coefficients are presented with 95% confidence intervals and corresponding p-values. b Mixed effect Model for patients with Spinal Anesthesia Mixed-effects linear regression model evaluating the association between time (wave), body mass index (BMI, centered), and hemodynamic response among patients receiving spinal anesthesia. Coefficients are presented with 95% confidence intervals and corresponding p-values. Supplementary file3 (DOCX 19 KB) [file 404_2026_8408_MOESM3_ESM.docx]

**Supplemental Table 3a Mixed effect model for patients with CSE**

|  | **Without Phenylephrine** | | | **With Phenylephrine** | | |
| --- | --- | --- | --- | --- | --- | --- |
| **Variable** | **Coefficient** | **95% CI** | **p-value** | **Coefficient** | **95% CI** | **p-value** |
| Wave | -1.8408 | (-2.0934, -1.5883) | <0.001 | -1.8408 | (-2.0933, -1.5882) | <0.001 |
| BMI (centered) | 0.231 | (0.0927, 0.3692) | 0.001 | 0.24 | (0.0968, 0.3832) | 0.001 |
| Wave × BMI | 0.0312 | (0.0005, 0.0619) | 0.046 | 0.0312 | (0.0005, 0.0619) | 0.046 |
| Wave (spline) | 1.1599 | (0.7815, 1.5383) | <0.001 | 1.1599 | (0.7815, 1.5382) | <0.001 |
| Wave (spline) × BMI | -0.0745 | (-0.1206, -0.0285) | 0.001 | -0.0745 | (-0.1205, -0.0285) | 0.001 |
| Hypertension | 0.4962 | (-1.7432, 2.7356) | 0.664 | 0.5009 | (-1.7382, 2.74) | 0.661 |
| Preeclampsia | 0.0111 | (-2.4815, 2.5037) | 0.993 | -0.022 | (-2.5178, 2.4738) | 0.986 |
| Gestational diabetes | 1.3381 | (-0.8693, 3.5455) | 0.235 | 1.34 | (-0.867, 3.547) | 0.234 |
| Phenylephrine | — | — | — | -0.3349 | (-1.7135, 1.0437) | 0.634 |
| **Constant** | 99.0427 | (97.7818, 100.3035) | <0.001 | 101.8446 | (90.2409, 113.4484) | <0.001 |

**Supplemental Table 3b Mixed effect Model for patients with Spinal Anesthesia**

|  | **Without Phenylephrine** | | | **With Phenylephrine** | | |
| --- | --- | --- | --- | --- | --- | --- |
| **Variable** | **Coefficient** | **95% CI** | **p-value** | **Coefficient** | **95% CI** | **p-value** |
| Wave | -1.8035 | (-2.017, -1.5901) | <0.001 | -1.8035 | (-2.017, -1.5901) | <0.001 |
| BMI (centered) | 0.2217 | (0.0857, 0.3578) | 0.001 | 0.2138 | (0.0749, 0.3528) | 0.003 |
| Wave × BMI | 0.0376 | (0.0066, 0.0686) | 0.017 | 0.0376 | (0.0066, 0.0686) | 0.017 |
| Wave (spline) | 1.063 | (0.7472, 1.3788) | <0.001 | 1.0632 | (0.7473, 1.379) | <0.001 |
| Wave (spline) × BMI | -0.0806 | (-0.1265, -0.0347) | 0.001 | -0.0806 | (-0.1265, -0.0347) | 0.001 |
| Hypertension | 0.0792 | (-1.9436, 2.1019) | 0.939 | 0.0823 | (-1.9402, 2.1047) | 0.936 |
| Preeclampsia | -1.7819 | (-3.8898, 0.3259) | 0.098 | -1.8004 | (-3.9089, 0.3082) | 0.094 |
| Gestational diabetes | 0.496 | (-1.411, 2.403) | 0.610 | 0.4835 | (-1.4236, 2.3907) | 0.619 |
| Phenylephrine | — | — | — | 0.3192 | (-0.8115, 1.45) | 0.580 |
| **Constant** | 100.1344 | (99.1141, 101.1547) | <0.001 | 97.5383 | (88.2852, 106.7915) | <0.001 |
